# Genome Sequence of *Kocuria varians* G6 Isolated from a Slaughterhouse in Denmark

**DOI:** 10.1128/genomeA.00076-16

**Published:** 2016-03-31

**Authors:** Prem K. Raghupathi, Jakob Herschend, Henriette L. Røder, Søren J. Sørensen, Mette Burmølle

**Affiliations:** Section for Microbiology, Department of Biology, University of Copenhagen, Copenhagen, Denmark

## Abstract

We report here the first draft genome sequence of *Kocuria varians* G6, which was isolated from a meat chopper at a small slaughterhouse in Denmark. The 2.90-Mb genome sequence consists of 95 contigs and contains 2,518 predicted protein-coding genes.

## GENOME ANNOUNCEMENT

*Kocuria varians* is a Gram-positive bacterium belonging to the family *Micrococcineae*. *Kocuria* species are ubiquitous and are highly adapted to their ecological niches (1). *K. varians* is predominantly isolated from different food-processing plants, e.g., milk-processing (2), fermented meat (3), and beef-processing plants (4). In these industrial settings, *K. varians* is often found in biofilms, and it is reported to favor both attachment and detachment of pathogens, namely, *Listeria monocytogenes* (2, 5). *K. varians* was also reported to cause brain abscess (6). Further, the beneficial effects of *K. varians* include improvement of the flavor profile in fermented sausage (7) and the degradation of putrescine (3). From a review of above-mentioned literature, *K. varians* has a central role in various complex interactions. Currently, there is no genome sequence information available for *K. varians*.

In this report, we announce the first draft assembly genome of *K. varians* isolated from a slaughterhouse in Denmark (8). The draft genome consists of 95 contigs, with an average G+C content of 70.5%. The whole-genome sequencing libraries were prepared using the Nextera XT kit (Illumina, USA), according to the manufacturer’s recommendations, and then sequenced as a part of the flow cell, as 2 × 250-base paired-end reads with Illumina MiSeq technology. The reads were cleaned and trimmed using CLC Genomics Workbench 7 (CLC bio, Denmark). The processed reads were assembled using SPAdes version 3.5.0 (9). The assembled genomes were uploaded to the RAST (10) server to perform functional annotations and to check and screen for noncoding rRNAs and tRNAs. rRNA genes were predicted by RNAmmer 1.2 (11).

The annotated results predicted 2,518 coding sequences (1,189 coding sequences [CDSs] have functional predictions), 50 RNA genes, 15 tRNA-coding genes, and 3 rRNA-coding genes. The genome has single predicted copies of 5S-16S-23S rRNA genes. The number of genes transcribed from the positive strand was 1,394, while 1,124 genes were transcribed from the negative strand. There are 364 predicted subsystems in the genome of G6, and we used this to reconstruct the metabolic network. The annotated genome has 22 genes involved in virulence, disease, and defense, including 10 genes coding for antibiotic resistance and 7 clustered regularly interspaced short palindromic repeat (CRISPR) elements, indicating the influence of phage exposure on the adaptation of this strain. Sixty-eight genes are involved in the stress responses of the bacterium. Functional comparison on the RAST server revealed the closest neighbors of *K. varians* to be *Kocuria rhizophila* DC2201, followed by *K. rhizophila* P7-4, *Rothia dentocariosa* ATCC 17931, and *Arthrobacter aurescens* TC1. Further work with this genome and comparisons to other *Kocuria* species will give more insights into the adaptation and evolution of *K. varians* to different environments.

### Nucleotide sequence accession numbers.

The whole-genome shotgun project for *K. varians* G6 has been deposited at the European Nucleotide Archive (ENA) under the contig accession numbers CZJX01000001 to CZJX01000095. The version described in this paper is the first version.
